# A Two-Step Approach for Classification in Alzheimer’s Disease

**DOI:** 10.3390/s22113966

**Published:** 2022-05-24

**Authors:** Ivanoe De Falco, Giuseppe De Pietro, Giovanna Sannino

**Affiliations:** Institute on High-Performance Computing and Networking (ICAR)—National Research Council of Italy (CNR), 80131 Naples, Italy; ivanoe.defalco@icar.cnr.it (I.D.F.); giuseppe.depietro@icar.cnr.it (G.D.P.)

**Keywords:** Alzheimer’s disease, magnetic resonance imagery, classification, interpretable machine learning, evolutionary algorithm

## Abstract

The classification of images is of high importance in medicine. In this sense, Deep learning methodologies show excellent performance with regard to accuracy. The drawback of these methodologies is the fact that they are black boxes, so no explanation is given to users on the reasons underlying their choices. In the medical domain, this lack of transparency and information, typical of black box models, brings practitioners to raise concerns, and the result is a resistance to the use of deep learning tools. In order to overcome this problem, a different Machine Learning approach to image classification is used here that is based on interpretability concepts thanks to the use of an evolutionary algorithm. It relies on the application of two steps in succession. The first receives a set of images in the inut and performs image filtering on them so that a numerical data set is generated. The second is a classifier, the kernel of which is an evolutionary algorithm. This latter, at the same time, classifies and automatically extracts explicit knowledge as a set of IF–THEN rules. This method is investigated with respect to a data set of MRI brain imagery referring to Alzheimer’s disease. Namely, a two-class data set (non-demented and moderate demented) and a three-class data set (non-demented, mild demented, and moderate demented) are extracted. The methodology shows good results in terms of accuracy (100% for the best run over the two-class problem and 91.49% for the best run over the three-class one), F_score (1.0000 and 0.9149, respectively), and Matthews Correlation Coefficient (1.0000 and 0.8763, respectively). To ascertain the quality of these results, they are contrasted against those from a wide set of well-known classifiers. The outcome of this comparison is that, in both problems, the methodology achieves the best results in terms of accuracy and F_score, whereas, for the Matthews Correlation Coefficient, it has the best result over the two-class problem and the second over the three-class one.

## 1. Introduction

Images are highly important in medicine for the diagnostic process. Through their examination, clinicians can draw hypotheses on whether or not a subject suffers from it and to what extent as well, so performing human-based classification.

Recently, automatic classification tools have been made available to clinicians by the advances in machine learning. Specifically, deep learning methodologies [1], consisting of Deep Neural Networks (DNNs), are, in practice, the standard for automatic image classification [2,3]. Through them, very high classification quality can often be achieved.

In spite of their excellent performance, DNNs are black boxes, i.e., no explanation is given by them on the reasons why a given item is assigned to a class. There is higher and higher interest, instead, in knowing these motivations. This is important, on the one hand, for experts who utilize DNN-based classification systems and, on the other hand, for individuals whose lives those decisions influence. This is the case, for example, in risk assessment problems, e.g., credit assignment in finance, recidivism risk prediction in court trials, and diagnosis in medicine. With reference to the medical domain, this lack of information typical of black box models can potentially have dangerous consequences and can even threaten lives. This brings practitioners to raise concerns, and the result is a resistance to the use of deep learning tools. These concepts of impediments caused by the black box nature, or opacity, of many machine learning algorithms, by their potentially deadly consequences, and by the need for model explanations in healthcare are well expressed, for example, in [4]. Instead, during the examination of medical images about a subject, doctors should be able to give motivations for their decisions and provide patients with explanations. In this case, an automatic classification system should be capable of persuading doctors by informing them of the reasons for that image being related to a positive or a negative case. At the same time, it should be able to reassure patients about the correctness of the decision and about the fact that their lives will not be threatened by it.

Given the above, it is easy to realize that a lot of research is being performed to design DNNs as well as other opaque (also termed black box) classification methodologies endowed with mechanisms for the explanation of their choices. This leads to *explainable* versions for Artificial Intelligence and Machine Learning [5] and lies in obtaining a posteriori another model able to explicitly tell users the motivations underlying its decisions. This takes place under the assumption that the behavior of the DNN’s internal model and that of this external one are totally the same for all the cases considered, as well as for new cases that might be shown in the future. Actually, this hypothesis is really strong. Cynthia Rudin [6] writes that “Explainable ML methods provide explanations that are not faithful to what the original model computes”. In fact, currently, the manners in which black boxes are endowed with explainability are, in many cases, unsatisfactory. She reports an example in [6], where a DNN is enriched with an attention mechanism and with saliency as the explaining model, yet this latter suggests that a specific item can equiprobabilistically represent both a Siberian husky and a transverse flute. In [6], she notes that “explanations often do not make sense, or do not provide enough detail to understand what the black box is doing”.

Unlike explainability, another way can be taken aiming at the creation of *interpretable* classification tools, where an explicit model can be directly created during the execution of the tool, so giving users explicit knowledge of the motivations by which items are classified. Different are the ways to represent this knowledge, for example, as decision trees or as rule sets. Actually, there is no unique definition of interpretability and of the meaning itself of the interpretation. Our view on interpretation is that, in it, explicit knowledge is extracted: in this paper, reference is made to the paper by Murdoch et al. [7], in which they state that `We define interpretable machine learning as the extraction of relevant knowledge from a machine-learning model concerning relationships either contained in data or learned by the model.’ Interpretable models are often criticized by saying that their performance is worse than that provided by explainable models. Yet, in [6], Rudin demonstrates that “It is a myth that there is necessarily a trade-off between accuracy and interpretability”.

The use of interpretable machine learning (IML) is rapidly gaining momentum in the scientific literature, and every year more and more such papers appear. Just to give some figures, on Web of Science, the number of articles related to IML per year goes from 154 in 2017 to 316 in 2018, and this number increases to 646 in 2019, 889 in 2020, up to 906 in 2021 [8]. This increase holds true when IML is applied to healthcare problems as well. Carrying out a review on this would be impossible here due to lack of space. Yet, some recent papers contain good reviews, on the one hand, on general, theoretical issues related to IML as taxonomy, medical application fields, challenges, and future directions, and, on the other hand, on lists and descriptions of practical applications. Among these papers, we can recall here at least [4] (2018), [9] (2020), [8] (2021), and [10] (2021).

## 2. Contribution and Organization

The present work makes use of a novel approach for the classification of images relying on interpretable ML. This approach is based on the use of an Evolutionary Algorithm (EA) to extract explicit knowledge useful for classification and is tested in the area of medicine, with particular regard to the diagnosis of Alzheimer’s disease [11].

Alzheimer’s disease is the commonest kind of dementia [12]. Generally speaking, the term ‘dementia’ is a broad word to describe a deterioration in intellectual capacity grave enough to disturb normal daily life and refers to a set of symptoms that negatively impact the brain: they cause difficulties in remembering, thinking clearly, making decisions, or even controlling emotions [13,14,15]. Different kinds and causes of dementia exist, e.g., vascular dementia, dementia with Lewy bodies, mixed dementia, frontotemporal dementia, Creutzfeldt–Jakob disease, Huntington’s disease, normal pressure hydrocephalus, and Alzheimer’s Disease. The latter is a specific type of dementia and is the most frequently encountered: around 60% to 80% of people with dementia have Alzheimer’s. It is a progressive brain disease that gradually leads to impairment in memory and cognitive function, worsens over time, and typically impacts subjects with ages from 65 on. The precise reason is uncertain, and no remedy is currently available. The most frequent early symptom is represented by problems in recalling events occurring recently. As the disease proceeds, further symptoms may appear, e.g., difficulties with speech, losing the sense of direction (also easily getting lost), frequent changes in mood, lack of enthusiasm, absence of self-care, and other problems related to behavior.

Given that this paper’s goal is to classify images related to Alzheimer’s disease, in the remaining of this paper, to avoid any possible confusion, wherever the terms ‘dementia’ or ‘demented’ are used, they always make specific and exclusive reference to Alzheimer’s disease. The use of these terms in the paper is due to the fact that they are utilized as class names in the original data set we have selected for our work.

One of the objective methods to assess the presence and the degree of Alzheimer’s disease in a patient lies in them undergoing a Magnetic Resonance Imaging (MRI) examination. Its outcome is a set of grayscale images that are examined by specialists so that they can deduce whether or not the disease affects that person and, if so, to what extent. This helps to assess if Alzheimer’s disease is at an initial stage or is progressing.

As far as we are aware, ours is the second paper in which Evolutionary Algorithms (EAs) are utilized for the classification over a data set of images, the first being [16], where we introduced the methodology and applied it to COVID-19 X-ray imagery. Particularly, this is the first time MRIs are considered. A wide literature survey by Nakane et al. [17] published in 2020 confirms this. In it, the authors firstly note the absence, in the last ten years or so, of recent surveys about the use of EAs and swarm algorithms for computer vision and images; hence their survey is the most recent and updated source. Even more importantly, they say that no paper reports on the use of EAs for the classification of images, whatever their type and source. It is known from the literature that the only use of EAs in the classification of images is to help find satisfactory DNN structures as well as the values for their hyperparameters [18,19]. Unfortunately, this has no effect on the fact that the DNN structures obtained behave as black boxes.

A two-step procedure is utilized here to classify images. The first step carries out pre-processing, meaning with this that each image is filtered and transformed into an array of real values, so obtaining a numerical data set starting from an image-based one. In the second stage, instead, the numerical data set obtained constitutes the input of an evolutionary-based interpretable classifier developed by us, the outputs of which are both the classification labels and automatically extracted explicit knowledge. This latter consists of a set of IF–THEN rules, each structured as a group of literals on the variables of the data set, all connected through AND logical operators.

The current work constitutes a preliminary study to ascertain whether the above method is both feasible and effective in tackling images related to Alzheimer’s disease. A free data set consisting of MR imagery related to Alzheimer’s disease has been downloaded and is used here, aiming to test if the medical domain can benefit from the approach and to which degree. In the paper, two experiments are reported by considering two and three classes, respectively.

The originality of our contribution to the scientific literature is twofold:

- firstly, a methodology is proposed for image classification that does not make use of the classically used Deep Neural Networks; this methodology is based on two steps, the second relying on an evolutionary-based classifier;
- secondly, this methodology based on an Evolutionary Algorithm is employed to classify MR images related to Alzheimer’s disease; as far as we know, no other paper in the literature describes the application of an EA to fulfill this task, which represents clear originality of this paper.

In the following of this paper, Section 3 outlines the related works. Section 4 illustrates the approach. Section 5 reports details on the data set utilized. Section 6 contains information about the experimental setup. Section 7 describes the experiments and the results on the two-class data set, as well as a comparison of the numerical results obtained against those achieved by a set of widely known well-performing classifiers. Section 8, instead, shows the same information with reference to the three-class data set. Section 9 contains a discussion of the pros and cons of the advanced approach. Finally, Section 10 summarizes conclusions and foreseen future actions.

## 3. Related Works

The application of Artificial Intelligence and Machine Learning methodologies has demonstrated very beneficial when dealing with images: in fact, these methodologies have proved extremely successful in tackling tasks such as, e.g., image segmentation, feature detection and selection, image matching, visual tracking, face recognition, human action recognition, and so on. A good and up-to-date survey on the use of EAs to deal with these tasks is given in [17].

As far as image classification task is considered, at the present time, deep learning structures represent the most advanced and widely used techniques, and Convolutional Neural Networks (CNNs) are the most frequently employed among them [20]. Researchers started using them in the 1980s; namely, in 1989, the first multilayered CNN called ConvNet was introduced by LeCun et al. [20]. After some initial interest, the drawback of high times for execution caused people to lose interest in them: in fact, cases were reported in which even small improvements in performance could only be obtained after weeks of execution. One of the reasons for that was the scarcity of both parallel computing methodologies and hardware needed for the training of that kind of networks. It was only in about 2010 that such practical limitations could be overpowered. In around the same years, improvements took place for the activation function when the originally employed Sigmoid was substituted with the Rectified Linear Unit or the Hyperbolic Tangent [21]. Lately, several more advancements have taken place, e.g., in the strategies to optimize the network parameters and in new concepts for architectural design, as, for example, in [22] (2018), [23] (2018), and [24] (2019).

In very recent times, lots of different such networks have been designed and are commonly utilized, among which, at least some should be mentioned here, as, e.g., LeNet [25] (1995), AlexNet [26] (2012), GoogleNet [27] (2015), ResNet [28] (2016), and DenseNet [29] (2017).

The focus of the present paper is on classical two-dimensional images. It is interesting to note here that when images in more than two dimensions are to be managed, other DNN methods have been developed for different image multi-modalities processing tasks: it is worth mentioning here at least dual-stream interactive networks for no-reference stereoscopic image quality assessment [30], viewport oriented graph convolution networks for blind omnidirectional image quality assessment [31], DNNs for 3D point cloud processing [32].

It should be mentioned here that CNNs are not the only Machine Learning algorithms being utilized to tackle the classification of images. In fact, the use of K-Nearest Neighbour [33], coupled with texture features, has proven successful in the discrimination of normal tissues from abnormal ones in medicine-related imagery [34] (2019). Further methods utilized to classify images include Support Vector Machines [35], Decision Trees [36], and shallow Artificial Neural Networks [37]. Regarding their performance, it is reported as good when the data set sizes are small or medium. However, it becomes worse than that provided by deep learning methodologies as soon as the size becomes large or very large, and this difference, in many cases, gets larger when the data sets contain higher numbers of classes.

In summary, CNNs exhibit good performance when classifying images, yet they show some limitations as well in this task. Firstly, CNNs work well when data sets are large, which allows good training; unfortunately, many image data sets are small. Transfer learning can help in decreasing the impact of this problem: in it, pre-training of the CNN takes place on a large data set containing images, and, once learning has been accomplished, this trained CNN can then be applied to the small image data set that is the real target of this classification task. Secondly, large amounts of memory and storage are required to run CNNs. Thirdly, as is the case for all Deep Neural Networks, at the end of the training, CNNs learn a black box model that relies on features implicitly extracted from the data, but such a model cannot be provided to an expert for them to check and approve it. It could be the case that classification could be misguided by those extracted features, which would yield unsatisfactory performance and, even worse, possible errors in doctors helping patients and risks to their health.

As a consequence, CNNs can profit from the use of techniques for extracting or selecting features. This task can be well accomplished through the use of meta-heuristic algorithms. With specific reference to images, feature selection has been carried out by different algorithms. Some examples include the utilization of a Genetic Algorithm (GA) over a data set containing nodules in lungs and breast [38] (2011), a Flower Pollination Algorithm to detect cancer in lungs [39] (2020), a Simulated Annealing scheme coupled to GA to classify brain tumors in MR imagery [40] (2019), a fuzzy Particle Swarm Optimization (PSO) scheme to deal with CT images showing examples of emphysema [41] (2019), a Bat Algorithm to tackle X-ray imagery of lungs [42] (2019), a hybrid algorithm composed of PSO coupled with fuzzy C-means to segment MR imagery [43] (2020), and an Artificial Bee Colony used for Parkinson’s disease [44] (2020). By looking at the dates of the publications just cited, it is evident that automatic feature selection in image classification is an important and still open problem.

Regarding Evolutionary Algorithms and Swarm Intelligence methodologies, a literature search reveals their use in the classification of images for some particular duties. A first task is represented by the automatic design of CNN structures, for which many examples exist, as in, e.g., [45] (2017), [18] (2018), [19] (2020), [46] (2020), [47] (2021), and [48] (2021) A second task is that of feature selection, for which EAs are used in, e.g., Ghosh [49] (2013), [50] (2015), [51] (2016), and [52] (2022).

As far as we know, there is no publication reporting on an EA being utilized to face on its own the task of image classification for Alzheimer’s disease. Hence, we believe that the present paper is innovative.

## 4. The Approach

**Figure 1:**
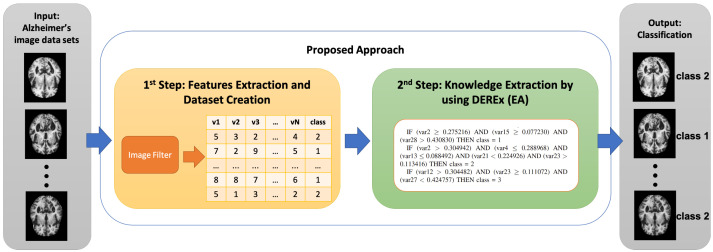
The approach. The left part of the image contains the filter (1st step), whereas the right part contains the classifier (2nd step) based on an Evolutionary Algorithm (EA). This latter also extracts explicit knowledge. MRI images contained in this figure are taken from the original Alzheimer’s data set [53] available on Kaggle.

Our approach relies on two steps being applied sequentially. Figure 1 displays that, as the first step, a data set composed of images is provided in the inut to a filter, the output of which is a numerical data set. As the second step, this latter is given as input to a classifying tool based on an Evolutionary Algorithm that, during its execution, also performs the automatic extraction of explicit knowledge. The two next subsections describe these two steps.

### 4.1. The Image Filter

Regarding the image filter, the one proposed by Mingjing Li [54] in the field of Context-based Image Retrieval has been chosen by us. The motivation for choosing that specific filter is that this filtering mode takes into account at the same time three different aspects related to the images: (i) the color moments of the image in the RGB color space; (ii) the texture moment of the image; (iii) the color correlogram in HSV color space. Thus, this filter is quite complete because it considers different issues. With greater detail, this filter encodes an input image in the form of an array with 64 real-valued attributes. These can be divided into three sets, each accounting for different kinds of features.

A first set consisting of six features refers to the two first color moments of the image in the RGB color space. The normalization of these values is effected through histogram normalization within [0.0, 1.0], so their sum is equal to 1.0.

A second set contains 14 attributes making reference to the texture moment of the image. Given the gray-level images of the faced data set, the feature extraction is in this paper restricted to the grayscale representation of the images. Because of this, rather than making reference to the color, these attributes in some way account for the structural information of the image. Namely, for each inner pixel present in the image, the filter calculates seven features that represent detected edge strengths at that pixel. The mean and variation for each attribute are computed over all the interior pixels. Normalization takes place here too, resulting in the sum of these values equalling 1.0.

A third set is composed of 44 attributes representing the color correlogram in HSV color space. This comprises the spatial correlation of image pixels. For their computation, a quantization of the HSV color space into 44 bins is performed, and, for each pixel, its auto correlogram is taken into account with its eight neighboring ones only. In HSV color space, the area that corresponds to black color is quantized into one bin, while the quantization for the gray levels, including white color, is performed with eight bins. For grayscale images, such as those used in this paper, only these nine bins will contain values different from zero. For this set too, value normalization takes place, so their sum is equal to 1.0.

Reference can be made to the original paper by Li [54] for more details about the meaning of these attributes, the reasons for their choice, or the motivations for their numbers.

This filter helps transform an image-based data set into a number-based one. The evolutionary-based classification algorithm will act on this latter.

### 4.2. The Evolutionary Classifying Tool

As the evolutionary-based classifying tool, it is utilized the Differential-Evolution-based Rule Extractor (DEREx) [55] that we designed and implemented. The motivation for choosing it is that, as far as we know, it is one of the very few ones that are based on Evolutionary Algorithms and that are specifically designed to automatically extract explicit knowledge from a numerical data set under the form of a set of IF–THEN rules. In [55], it was compared over a set of medical data sets against many other Machine Learning classification methodologies and turned out to be the best in terms of accuracy. Moreover, we designed it, so its use was straightforward to us. DEREx is a classification algorithm, the basic component of which is a Differential Evolution (DE) algorithm [56,57], a particular EA suited to multi-variable numerical optimization. In DEREx, the general DE scheme is endowed with a wide set of features to make it suitable for a classification problem.

#### 4.2.1. Differential Evolution

DE constitutes an optimization procedure used to face real-valued problems and is based on imitating in a computer the evolution of a population of individuals happening in nature. Shortly, when a real-valued problem needs optimization, DE starts by randomly creating an initial set, called *population*, of solutions that are named *individuals*, let their number be *Pop_Size*, and computes for each of them the value of an objective function named *fitness*. This latter represents the goodness of the solution under account at solving the given problem. If the numerical problem to be optimized has $N_{par}$ parameters, each individual in DE is an array of $N_{par}$ real values. Then, DE is an iterative procedure: for a given number of iterations *Max_Gens*, each of which is called a *generation*, DE modifies the set of the currently available solutions; for each current solution, a new trial one is generated thanks to the use of two operators, called crossover and mutation, and the fitter between them is added to the next population. The execution of DE depends on a set of parameters, among which are the crossover ratio CR and the mutation factor FV. These latter are involved in the way the current solutions are utilized in the creation of new ones. These parameter values impact the evolution and, therefore, the final best solution obtained. Details on DE may be found in the seminal papers [56,57].

Ten different strategies to generate a trial individual have been designed in DE. Just to provide an example, here, the strategy referenced as *DE/rand-to-best/1/bin* is described because it has been used in the experiments reported in this paper. In it, to create a new population, for the generic *i*-th individual in the current population, two integer numbers $r_{1}$ and $r_{2}$ in [1, …, *Pop_Size*] differing from each other and different from *i* are randomly generated. Furthermore, another integer number *s* in the range [1, $N_{par}$] is randomly chosen. Then, starting from the *i*-th individual, a new trial one $i^{\prime }$ is generated whose generic *j*-th component is given by

(1) $$x_{i^{\prime },j}=x_{i,j}+FV\cdot \left(best_{j}-x_{i,j}\right)+FV\cdot \left(x_{r_{1},j}-x_{r_{2},j}\right)$$

provided that either a randomly generated real number ρ in [0.0,1.0] is lower than CR or the position *j* under account is exactly *s*. If neither is verified, then a simple copy takes place: $x_{i^{\prime },j}=x_{i,j}$. FV is a real and constant factor that controls the magnitude of the differential variation $\left(x_{r_{1},j}-x_{r_{2},j}\right)$ and is a parameter of the algorithm, and $best_{j}$ is the *j*-th component of the current best individual in the population.

The general DE scheme is shown in Algorithm 1 for a maximization problem as that faced in this paper.
**Algorithm 1** DE Algorithm**begin**   **randomly initialize** the population   **evaluate** the fitness Φ of all the individuals   **while** (maximal number of generations Max_Gens is not reached) **do**     **begin**       **for** i=1 **to** Pop_Size **do**          **begin**            **create** a new trial individual $x_{i^{\prime }}$ by using one of the ten DE strategies            **if**              $\Phi \left(x_{i^{\prime }}\right)\geq \Phi \left(x_{i}\right)$                **insert** $x_{i^{\prime }}$ in the new population              **else**                **insert** $x_{i}$ in the new population          **end**     **end****end**

In Algorithm 1, $x_{i}$ represents the generic *i*-th individual in the current population, and $\Phi (x_{i})$ is its fitness value. Moreover, $x_{i^{\prime }}$ is the trial individual that could replace the current *i*-th in the next population being created, and $\Phi (x_{i^{\prime }})$ is its fitness value.

#### 4.2.2. DEREx

Particularizing from the general DE scheme to DEREx, let us suppose that we have to classify items in a data set with $N_{V}$ attributes and $N_{C}$ classes. Each solution in DEREx is, then, a real-valued vector encoding a set of *N_Max_Rules* classification rules (*N_Max_Rules* is a parameter for the DEREx algorithm).

In order to encode a set of *N_Max_Rules* rules, each over a maximum of $N_{V}$ attributes, the length $N_{G}$ of each real-valued solution vector in DEREx will be equal to

(2) $$N_{G}=N\_Max\_Rules\times \left(1+\left(4\times N_{V}\right)+1\right)$$

The explanation of this length would require too much space here and can be found in [55].

Each of these rules has the form IF (condition) THEN (class). The conditional part of any rule consists of a set of literals connected through AND logical operators. Each such literal has the following form:

$$\left(var_{i}\;OP\;const_{1}\;const_{2}\right)$$

where $var_{i}$ represents one of the $N_{V}$ data set attributes, $const_{1}$ and $const_{2}$ are two numerical constants, while OP represents a relational operator. This can be one among <,≤,≥,>,IN,OUT. The first four operators need one constant, so $const_{1}$ only is used in these literals, while the last two mean that $var_{i}$ is in a given range or outside it, respectively; therefore, these literals need both $const_{1}$ and $const_{2}$.

The greatest number of rules that can constitute a rule set is designated by *N_Max_Rules*. Actually, each such rule may or may not be active and participate in the classification process. This depends on the value of another DEREx parameter called ’rule threshold’ (*Rule_Thr*) that can also be set by users in [0.0–1.0]: the lower this value, the less probably a rule set will be composed of less than *N_Max_Rules* rules. Concerning the number of literals active in each rule in the generic solution, another DEREx parameter called ’literal threshold’ *Lit_Thr* must be set by the user in [0.0–1.0]: the lower its value, the higher the number of literals a rule contains. Through the use of these parameters, users can adjust both the expected number of the rules in a solution that are active in the classification process and their expected length in terms of the number of active literals.

As an example of the use of DEREx to classify over a two-class data set containing five attributes, this algorithm could propose a solution as:

IF (var2 < 0.78) AND (var3 < 0.12) THEN class = 2

IF (var3 ≥ 0.08) AND (var1 > 0.37) THEN class = 1

IF (var5 OUT (0.36, 0.54)) THEN class = 2

When an item has to be assigned to a class, three different situations can occur. In the first situation, the item is caught by just one rule or by more rules, all suggesting the same class: in this situation, the item is allocated to that class. In the second situation, the item is caught by more rules suggesting differing classes: it cannot be directly assigned to a class. This is termed a *yes–yes* indeterminate case. In the third situation, the item is caught by no rule: in this case too, it cannot be directly assigned to a class. This is termed a *no–no* indeterminate situation.

DEREx contains a suitable procedure allowing users to positively solve both *yes–yes* and *no–no* indeterminate situations; thanks to it, DEREx can assign each item to just one class.

Similar to the general DE scheme, DEREx allows choosing one from among ten possible evolution algorithms; let us denote it with *DE_Algo*. The differences among these schemes rely on how a new trial solution is obtained starting from the currently available ones.

The seminal paper [55], in which we designed and implemented DEREx, provides all the details on all of the above.

## 5. The Data Set

The original Alzheimer’s data set [53] was downloaded from Kaggle. It was originally assembled in 2020 by Sarvesh Dubey, who hand-collected images and labels from various websites. This data set consists of MRI images of brains, and the aim is to discriminate the presence or absence of Alzheimer’s disease and, in the positive case, to assess its stage. The images were also segmented by the data set creators, and, as of today, the data set has been downloaded 9422 times, so it is widely known and used in the literature in recent papers, as in, e.g., [58,59,60].

With reference to this data set, we have started by taking into account all those items corresponding to the highest level of Alzheimer’s disease, there labeled as *moderate demented*. They are very few, just 52. In order to discriminate this class against the cases in which the disease is absent, reference has been made to the *non-demented* class. This contains 2560 items, which would yield a data set too unbalanced. Therefore, just the first 68 items from the non-demented class have been considered, and a quite well-balanced data set with 120 images to be assigned to two classes has been created.

In order to create a more difficult case, the intermediate *mild demented* class has been considered so as to create a three-class scenario. In this case, 52 items for each of the three classes have been considered for a total of 156 items.

**Figure 2:**
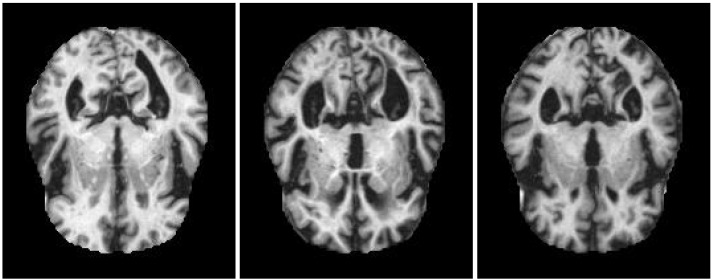
Example of items from the three classes. **Left pane**: *non-demented*. **Center pane**: *mild demented*. **Right pane**: *moderate demented*. MRI images contained in this figure are taken from the original Alzheimer’s data set [53] available on Kaggle.

Some examples of the three classes are shown in Figure 2. At first sight, the images belonging to different classes are much the same in both color and shape, and their dissimilarities appear slight. Nonetheless, an expert clinician can spot the areas containing differences and, hence, assign the images to the different classes.

## 6. Experimental Setup

The experiments take into account two different situations. The first is easier and considers just two classes, namely, the two extreme cases *non-demented* and *moderate demented*. The second, instead, is more difficult, and three classes are considered in it. The presence of the intermediate class could make things more complicated, as the differences between classes may now be quite fuzzier than before.

To set our system, we have had to make some decisions. Firstly, we utilize here a filter tool publicly available on GitHub [61], thanks to Xirong Li and his colleagues, who applied it in a neighbor voting algorithm [62]. Differently from Mingjing Li’s paper, Xirong Li et al. chose a different order for the features: they put the 44 correlogram features first, followed by the 14 related to texture moment, the six color moment features being in the last positions.

Another important issue about the filtering tool is that grayscale images are dealt with here. Consequently, use is not made here of some of the 64 features in the original tool by Mingjing Li. Actually, 35 of them always have values of zero in each data set item. This is to be expected, as all the correlogram features referring to non-gray colors hold null values in color-related bins, whereas the only non-empty bins are those referring to black, gray, and white levels, i.e., a total of nine non-empty attributes. Given this, and aiming to avoid DEREx investigating a wider search space containing a great amount of physically unviable solutions, the cardinality of the considered features has been reduced here from 64 down to 29.

In Table 1, the encoding with the 29 features and their positions is reported, and a brief description of their meaning is given.

Regarding the classification, instead, it is to be pointed out that DEREx is a stochastic algorithm: its execution relies on the value of a random seed to be chosen before running DEREx. To get rid of this, for each classification task DEREx has been executed 25 times by using 25 differing seeds. Thanks to this, different evolutions have been obtained, resulting in different final solutions.

To quantify the classification quality of each solution, the accuracy $A_{cc}$ has been used as the *fitness* function:

(3) $$A_{cc}=\frac{CC}{N_{items}}\times 100.0\%$$

where CC represents the number of items faultlessly classified, and $N_{items}$ represents the total amount of items. $A_{cc}$ values range in [0.0, 100.0], where a higher value implies a more accurate classification, so this is a maximization task.

Other indices that are widely used to evaluate the quality of classifications obtained and to compare those provided by different algorithms are the F_score and the Matthews Correlation Coefficient (MCC). We will consider them too.

To define them for a binary classification problem in the medical domain, firstly, we take as the positive class the one containing the items related to the disease, the negative one being that with the items of the non-disease class. Then, given a classification obtained on the data set items, we can divide them as:

- true positive (*tp*): the positive items correctly assigned to the positive class;
- true negative (*tn*): the negative items correctly assigned to the negative class;
- false positive (*fp*): the negative items wrongly assigned to the positive class;
- false negative (*fn*): the positive items wrongly assigned to the negative class.

With these definitions, the F_score index is defined as:

(4) $$F\_score=\frac{tp}{tp+\frac{1}{2}\cdot \left(fp+fn\right)}$$

while the MCC index is defined as:

(5) $$MCC=\frac{\left(tp\cdot tn)-(fp\cdot fn\right)}{\sqrt{\left(tp+fp)\cdot (tp+fn)\cdot (tn+fp)\cdot (tn+fn\right)}}$$

These definitions can be extended to data sets with more than two classes.

The admissible range for the F_score is [0.0, 1.0], while that for MCC is [−1.0, 1.0]: the higher these values, the better the classification.

For both the data sets described above, the goal is to effect supervised learning; therefore, the items are split into two sets: a train set, with the first 70% of the items in their sequential order, and a test set with the last 30%.

Out of the 25 runs, that for which the highest classification accuracy is obtained over the train set is considered the best one, and the performance of that run is appraised through the accuracy value obtained over the unseen test set items. Hence, the results considered could not correspond to the highest value obtained by DEREx over the test set.

The next two sections report on the findings for the two diverse scenarios, starting from the two-class task.

To run the experiments, we have written an implementation of DEREx in C language and have utilized a Mac Pro to run it. This latter runs MacOS High Sierra as the operating system and, from a hardware point of view, is equipped with two 3.5 Ghz Intel Xeon E5 processors, each with six cores, a 256 kB L2 cache per core, 32 GB DDR3 ECC memory, and a 1 TB storage disk.

## 7. Two-Class Classification

### 7.1. Settings

In this case, Table 2 shows the settings used for the parameters of DEREx. No preparatory phase has been carried out to set those values; rather, the typical setting utilized for two-class classification has been used.

As explained in Section 4.2.2, these values mean that we desire to obtain rule sets consisting of just two rules (one for each class) (*N_Max_Rules* = 2 and *Rule_Thr* = 0.0), each containing a small number of literals (*Lit_Thr* = 0.90). This shows our wish to achieve compact and easily legible knowledge, even if this should imply a slight decrease in the accuracy.

The way in which the class names are represented by integer values is shown in the last two rows of Table 2.

Given that the filtering step outputs the numerical data set in a way that its items are sequentially grouped class by class, a random shuffle takes place on them, after which the train and test sets are assigned the first 84 and the last 36 items, respectively.

### 7.2. Results

DEREx shows excellent performance on this problem. In fact, the average value for $A_{cc}$ achieved on the train set over the 25 executions is 99.00%. Moreover, the average $A_{cc}$ value over the test set is 96.89%. This proves that the algorithm has excellent generalization ability because it misclassifies, on average, just one or two items of the previously unseen test set items. So, the classifier has actually comprehended the differences existing between the classes.

As said above, the best run is the one obtaining the highest $A_{cc}$ value on the training set. It obtains $A_{cc}$ values of 100% over both the train and the test sets, so it never makes mistakes. Of course, the resulting $A_{cc}$ value over the total data set is 100%. In Table 3, the confusion matrices for the best solution over the train set, the test set, and the total data set are shown.

The best two-rule solution achieved is:

IF (var13 < 0.090) AND (var23 > 0.114) THEN class = 1

IF (var5 > 0.195) AND (var11 ≤ 0.374) AND (var17 OUT (0.135 0.145)) THEN class = 2

It contains just five parameters out of the 29. For class 1 (*moderate demented*), the rule is very compact and easily legible because it comprises just two parameters, both related to the texture moment attributes, more specifically related to two intermediate gray bins.

### 7.3. Comparison

The Waikato Environment for Knowledge Analysis (WEKA) [63] tool, version 3.8.5, has been utilized to run other classifiers on the two-class data set. This tool contains a large number of classification algorithms that may be split into groups: all the algorithms in a given group are based on very similar ideas and working mechanisms. Just to give some examples, a group is made up of classification methodologies based on bayesian ideas, another group collects classifiers relying on functions, yet another contains algorithms using classification trees, and so on. In Table 4, the methodologies utilized here are shown. For each such methodology, the table reports several pieces of information: in the first column, the class of the method is reported; in the second, its name is given; in the third, the acronym used for it is contained; and in the last, a useful citation about the method is given for interested readers.

Each of the methodologies shown in Table 4 has its own parameter set. Setting good values for all these parameters is an important step for the execution of each such algorithm. To carry out the experiments reported in this paper, we have decided to run each of them by utilizing the default parameter values as these are assigned in WEKA. This is because a high amount of time would be needed to perform a preliminary phase for the tuning of the values of these parameters, and this should be repeated for each algorithm considered here. In these conditions, the comparison with DEREx is fair because we have not tuned the values of the DEREx parameters. Moreover, similarly to DEREx, each algorithm has been run 25 times by varying the value of the random seed parameter, where available. The same division of the items into a train set and a test set that has been used for DEREx has been utilized here too.

Table 5 shows the results in terms of accuracy, F_score, and MCC achieved over the test set by all the algorithms, DEREx included. For each such index, both the average of the 25 final values and the best value among the 25 are given. For each parameter, the result of the best-performing algorithm is reported in bold.

In general, the results show that, for each of the three performance indices, each algorithm run within WEKA always reaches the same final value, be it the best possible or not, and this holds true independently of the initial random seed. With reference to the three indices, DEREx obtains the perfect solution characterized by 100.00% the performance for accuracy and 1.0 for both F_score and MCC. Apart from DEREx, there are other six algorithms finding solutions leading to perfect classification, i.e., 100% of accuracy or 1.0 for F_score and MCC over the test set. These results imply that this problem is quite easy for many of the classifiers investigated.

## 8. Three-Class Classification

### 8.1. Settings

For this scenario, instead, Table 6 shows the parameter setting used. For this problem too, no preparatory phase to tune the values of DEREx parameters has been performed. The values of *Pop_Size* and *Max_Gens* have been empirically increased because this problem seems more difficult than the former, and the search space is larger due to the presence of three classes. Of course, the value for *N_Max_Rules* has been increased up to three.

In this case too, the values for *N_Max_Rules* and *Rule_Thr* imply that we are looking for a set of three rules, one per class, while that for *Lit_Thr* pushes towards compact rules.

The way in which the names of the three classes are represented by integer values is shown in the last three rows of Table 6.

Here too, random shuffling has been done, and the train set has been filled with 109 items while the test set with 47.

### 8.2. Results

DEREx shows good performance on this problem too. In fact, the average value for $A_{cc}$ achieved on the train set over the 25 executions is 89.06%. Moreover, the average $A_{cc}$ value over the test set is 86.21%. The figures are lower than those in the two-class problem; hence they confirm that this latter problem is more difficult because the differences between the three classes are now fuzzier.

The best run achieves $A_{cc}$ values of 90.83% on the train set, 91.49% over the test set, and 91.03% over the whole data set. In Table 7, the confusion matrices for the best solution over the same three sets are shown in the same order as above.

As a general comment to the table, all the items in class 1 (*mild demented*) are correctly taken. Regarding the items of class 2, i.e., the *moderate demented*, for three of them, a wrong assignment to the class of the *mild demented* takes place, as could be expected. Finally, for class 3 (*non-demented*), the same takes place, although more frequently. Just an item from *non-demented* is wrongly assigned to *moderate demented*.

The best three-rule solution achieved is:

IF (var3 ≤ 0.299) AND (var9 < 0.368) AND (var10 IN (0.076 0.079)) AND (var12 ≤ 0.293) AND (var15 ≥ 0.078) AND (var18 < 0.552) THEN class = 1

IF (var5 < 0.117) AND (var13 < 0.085) THEN class = 2

IF (var26 ≥ 0.392) THEN class = 3

In this case, three rules are enough to obtain good performance. Nine parameters are contained out of the 29. In addition, all three different types of attributes are contained in the rule set.

### 8.3. Comparison

Here too, the same classifiers in WEKA utilized in the two-class problem have been run. The operating conditions are the same as described in Section 7.3.

Table 8 reports for each classifier the same pieces of information taken into account for the two-class problem, i.e., average and best values for accuracy, F_score, and MCC. Here too, the value obtained by the best-performing algorithm is displayed in bold.

An overall observation about the contents of the table is that all the investigated algorithms present lower values than those for the two-class problem; hence this proves that this problem is more difficult than the previous one.

The analysis of the algorithms, in this case, shows that, for accuracy, DEREX reaches the highest best value together with MLP and Random Forest. The same takes place for F_score at parity with MLP. For MCC, instead, DEREx is the runner-up, being only second to MLP. The advantage of DEREX over its two closest competitors is given by the simplicity of its explicit rules. In fact, MLP creates a black box model that cannot provide doctors with any explicit and easy-to understand information, which does not help with the issue of trustworthiness. Random Forest, in its turn, creates for this classification problem very complicated tree structures that are hard to follow for humans. Given the above, we do feel that the use of DEREx is preferable in terms of the trustworthiness and simplicity of the solution.

## 9. Discussion

Below, we shortly discuss the strengths and the weaknesses of the methodology.

As a first pro, it should be observed that images belonging to different classes are actually quite alike, as Figure 2 has shown. Adding to this, the colors of the images from diverse classes are practically equal. Hence, our approach cannot rely on differences in colors or shapes when assigning the items to the classes. With regards to this, these experiments show a worst-case situation. Nonetheless, this does not impact results.

A second pro is that this approach considers colors, so it seems promising for the situations where the typical items of different classes are in dissimilar colors. As an example, a set of preliminary experiments on bird classification over the CUB_200_2011 data set [78,79] has allowed us to obtain very high accuracy values when distinguishing among birds such as common yellowthroat, red-cockaded woodpecker, and red-headed woodpecker. That three-class data set contains around 60 items per class. The figures achieved for $A_{cc}$ over train and test sets are equal to 97.52% and 96.23%, respectively. These figures suggest that, when images are colored, an improvement in classification accuracy takes place with respect to grayscale situations.

A third pro involves the amount of knowledge that is extracted by DEREx and is needed to perform classification. All the rule-based and the tree-based algorithms considered in this paper also perform interpretable machine learning; in fact, they all directly extract explicit knowledge and provide it to the user under the form of rule sets and decision trees, respectively. Consequently, a comparison can be carried out on the amount of information extracted by both DEREx and them to achieve the numerical performance shown in the previous tables. To this aim, we can consider here, for each rule-based algorithm and for each problem, the number of the rules needed by the best solution found and the number of the literals contained in them for each problem. Instead, as far as tree-based algorithms are taken into account, we consider for each problem the number of tree leaves rather than that of the rules, whereas the number of literals has the same meaning as in the previous case. In the following, we use $n_{r}$ to represent the number of rules for the rule-based algorithms and that of leaves for the tree-based ones, whereas $n_{l}$ denotes, in both cases, the number of literals. Table 9 shows, for each algorithm and for each problem, those two values. Furthermore, the three last columns of the table report the average numbers of rules/leaves $\langle n_{r}\rangle$ and of literals per problem $\langle n_{l}\rangle$ needed by each technique, and also the average number of literals per rule/leaf $\langle n_{l/r}\rangle$.

As it can be seen, on average, DEREX requires the lowest number of rules/leaves, apart from, of course, OneR that, for definition, only creates one rule, which could somehow limit its performance. The lowest number of literals per problem is, instead, achieved by JRip, whereas DEREx requires a number of literals higher, yet about half of the one required by its closest competitor in terms of classification performance, i.e., Random Forest. As far as the number of literals per rule/leaf is considered, it can be seen that DEREx has the second-highest value. In summarizing, DEREx needs few rules and fewer literals to obtain performance equivalent to that of the well-performing Random Forest and better than that of the other classifiers providing explicit knowledge. Hence, it provides compact and easy-to-understand knowledge that helps to correctly classify, so its use can be well suited in the medical field to support doctors in making their decisions.

A first weakness of the experiments reported here is the small data set size. From a practical viewpoint, frequently, doctors only have a few dozens of images available. Yet, an investigation should be conducted about the robustness of the approach as the data set dimension varies.

A second weakness may consist in this data set being well balanced among the classes. An investigation should be performed on how this approach behaves when the data set classes are largely unbalanced. In such cases, quality indices such as $F_{score}$ or MCC are better suited than $A_{cc}$.

In addition, an investigation should take place when diverse kinds of image data sets are considered, with reference to data sets in which typical items belonging to the same class are in dissimilar colors or items of different classes have the same color.

Last but by no way least, from the perspective of interpretability, looking for other filters providing other parameters of high significance for us humans is an activity worth investigating. As an example, they could give information concerning a specific area of the image: “this specific part of the image suggests that …”.

## 10. Conclusions and Future Works

A methodology has been employed that we designed, and that is based on two steps to classify images, based both on Context-based Image Retrieval ideas and on an evolutionary tool automatically extracting explicit knowledge. This methodology has been tested on an MRI image data set referring to Alzheimer’s disease.

The outcomes achieved seem promising, and the approach does not show evident problems, yet wider research should be effected to investigate its efficiency on other data sets, with specific reference to the medical field. The discussion in Section 9 has outlined some guidelines for future work, with specific reference to the general applicability of the methodology. The filtering step is of high interest regarding the number of attributes for each of its three components. Several questions arise with respect to this: are more orders for the color moment beneficial? Does a number of bins for the color correlogram other than 44 yield higher performance? Can other texture moments be considered? Investigations will be performed on these issues.

For our future work, concerning interpretability, we will seek further filters providing parameters of high significance for us humans, e.g., those providing information on a specific image area. Moreover, an important issue related to dementia lies in investigating where the brain loss is in the MRI images rather than in merely categorizing the images. Unfortunately, this has not been possible in the present paper because the data set used only refers to Alzheimer’s disease; moreover, it does not contain information on areas with brain loss. Therefore, in further future work, a search will be performed in the literature to find data sets about dementia rather than simply on Alzheimer’s disease. The aim will be to check the ability of our methodology to distinguish among different dementia types and investigate where the brain loss is in the MRI images.

## Figures and Tables

**Table 1:** The encoding with the 29 attributes and their positions.

*Number of the Attribute*	*Description of the Attribute*
1	color correlogram: black bin
2 to 8	color correlogram: gray bins
9	color correlogram: white bin
10–16	mean values of the seven texture moment attributes
17–23	variation values of the seven texture moment attributes
24	first-order R color moment
25	first-order G color moment
26	first-order B color moment
27	second-order R color moment
28	second-order G color moment
29	second-order B color moment

**Table 2:** The Parameter Setting for DEREx for the 2-Class Problem.

*Parameter*	*Setting*
Pop_Size	30
Max_Gens	500
Cr_Ratio	0.3
Mut_F	0.75
DE_Algo	DE/rand-to-best/1/bin
N_Max_Rules	2
Rule_Thr	0.0
Lit_Thr	0.90
class 1	*moderate-demented*
class 2	*non-demented*

**Table 3:** Confusion Matrices of the Best Rule Set (2-Class Problem).

	*Train Set*		*Test Set*		*Whole Data Set*	
	***Predicted Class***		***Predicted Class***		***Predicted Class***	
***Real Class***	**1**	**2**	**1**	**2**	**1**	**2**
**1**	37	0	15	0	52	0
**2**	0	47	0	21	0	68

**Table 4:** The classification algorithms contained in WEKA and used in this paper.

Class	Algorithm	Acronym	Reference
*Bayes:*	Bayes Net	BN	[64]
	Naive Bayes	NB	[65]
*Functions:*	MultiLayer Perceptron	MLP	[66]
	Radial Basis Function	RBF	[67]
	Support Vector Machine	SVM	[68]
*Meta:*	AdaBoost	AB	[69]
	Bagging	Bag	[70]
*Rules:*	One Rule	OneR	[71]
	Repeated Incremental Pruning (JRip)	JRip	[72]
	Partial Decision Tree (PART)	PART	[73]
	Ripple-Down Rule	Ridor	[74]
*Trees:*	C4.5 decision tree (J48)	J48	[75]
	Random Forest	RF	[76]
	REPTree	RT	[77]

**Table 5:** The numerical results for the 2-Class Problem.

	*Accuracy*		FF_Score		*MCC*	
	Average	Best	Average	Best	Average	Best
Bayes Net	97.22 %	97.22%	0.9721	0.9721	0.9439	0.9439
Naive Bayes	97.22%	97.22%	0.9721	0.9721	0.9439	0.9439
MLP	97.22%	97.22%	0.9724	0.9724	0.9439	0.9439
RBF	**100.00%**	**100.00%**	**1.0000 **	**1.0000**	**1.0000**	**1.0000**
SVM	**100.00%**	**100.00%**	**1.0000**	**1.0000**	**1.0000**	**1.0000**
Adaboost	**100.00%**	**100.00%**	**1.0000**	**1.0000**	**1.0000**	**1.0000**
Bagging	94.44%	94.44%	0.9448	0.9448	0.8935	0.8935
JRip	94.44%	94.44%	0.9448	0.9448	0.8935	0.8935
OneR	94.44%	94.44%	0.9438	0.9438	0.8896	0.8896
PART	**100.00%**	**100.00%**	**1.0000**	**1.0000**	**1.0000**	**1.0000**
Ridor	88.89%	88.89%	0.8896	0.8896	0.7994	0.7994
J48	**100.00%**	**100.00%**	**1.0000**	**1.0000**	**1.0000**	**1.0000**
Random Forest	**100.00%**	**100.00%**	**1.0000**	**1.0000**	**1.0000**	**1.0000**
REPTree	88.89%	88.89%	0.8896	0.8896	0.7994	0.7994
DEREx	96.89%	**100.00%**	0.9683	**1.0000**	0.9385	**1.0000**

**Table 6:** The Parameter Setting for DEREx for the 3-Class Problem.

*Parameter*	*Setting*
Pop_Size	50
Max_Gens	5000
Cr_Ratio	0.3
Mut_F	0.75
DE_Algo	DE/rand-to-best/1/bin
N_Max_Rules	3
Rule_Thr	0.00
Lit_Thr	0.90
class 1	*mild demented*
class 2	*moderate demented*
class 3	*non-demented*

**Table 7:** Confusion Matrices of the Best Rule Set (3-Class Problem).

	*Train Set*			*Test Set*			*Whole Data Set*		
	***Predicted Class***			***Predicted Class***			***Predicted Class***		
***Real Class***	**1**	**2**	**3**	**1**	**2**	**3**	**1**	**2**	**3**
**1**	35	0	0	17	0	0	52	0	0
**2**	2	38	0	1	11	0	3	49	0
**3**	8	0	26	2	1	15	10	1	41

**Table 8:** The numerical results for the 3-Class Problem.

	*Accuracy*		F_Score		*MCC*	
	Average	Best	Average	Best	Average	Best
Bayes Net	85.11 %	85.11%	0.8491	0.8491	0.7748	0.7748
Naive Bayes	74.47%	74.47%	0.7351	0.7351	0.6080	0.6080
MLP	**91.49%**	**91.49%**	**0.9149 **	**0.9149**	**0.9144**	**0.9144**
RBF	76.60%	76.60%	0.7604	0.7604	0.6382	0.6382
SVM	87.23%	87.23%	0.8722	0.8722	0.8023	0.8023
Adaboost	85.11%	85.11%	0.8508	0.8508	0.7718	0.7718
Bagging	89.36%	89.36%	0.8936	0.8936	0.8312	0.8312
JRip	89.36%	89.36%	0.8950	0.8950	0.8389	0.8389
OneR	72.34%	72.34%	0.7021	0.7021	0.6003	0.6003
PART	82.98%	82.98%	0.8298	0.8298	0.7346	0.7346
Ridor	89.36%	89.36%	0.8928	0.8928	0.8384	0.8384
J48	82.98%	82.98%	0.8261	0.8261	0.7350	0.7350
Random Forest	**91.49%**	**91.49%**	0.9137	0.9137	0.8682	0.8682
REPTree	87.23%	87.23%	0.8738	0.8738	0.8086	0.8086
DEREx	86.21%	**91.49%**	0.8675	**0.9149**	0.7902	0.8763

**Table 9:** Size of information extracted by the classifiers performing interpretable machine learning.

	Two-Class		Three-Class				
	$n_{r}$	$n_{l}$	$n_{r}$	$n_{l}$	$\langle n_{r}\rangle$	$\langle n_{l}\rangle$	$\langle n_{l/r}\rangle$
JRip	3	2	4	5	3.50	3.50	1.00
OneR	1	2	1	7	1.00	4.50	4.50
PART	3	2	8	16	5.50	9.00	1.64
Ridor	3	3	3	3	3.00	3.00	1.00
J48	3	4	14	27	8.50	15.50	1.82
Random Forest	4	6	13	25	8.50	15.50	1.82
RepTree	2	2	4	9	3.00	5.50	1.83
DEREx	2	8	3	7	2.50	7.50	3.00

## Data Availability

Alzheimer’s Dataset used in this study is available online: https://www.kaggle.com/tourist55/alzheimers-dataset-4-class-of-images (accessed on 30 March 2022).
